# Pathological and Molecular Characterization of a Duck Plague Outbreak in Southern China in 2021

**DOI:** 10.3390/ani12243523

**Published:** 2022-12-13

**Authors:** Zhipeng Liang, Jinyue Guo, Sheng Yuan, Qing Cheng, Xinyu Zhang, Zhun Liu, Congying Wang, Zhili Li, Bo Hou, Shujian Huang, Feng Wen

**Affiliations:** 1 College of Life Science and Engineering, Foshan University, Foshan 528231, China; 2 Institute of Animal Husbandry and Veterinary Medicine, Fujian Academy of Agricultural Sciences/Fujian Animal Disease Control Technology Development Center, Fuzhou 350013, China; 3 Guangdong Provincial Key Laboratory of Animal Molecular Design and Precise Breeding, College of Life Science and Engineering, Foshan University, Foshan, 528231, China

**Keywords:** duck plague, duck plague virus, *Herpesviridae*, *UL2*, *LORF11*

## Abstract

**Simple Summary:**

This study reports the pathology, molecular detection, isolation, and genetic characterization of a novel duck plague virus from a recent outbreak affecting domestic layer ducks in southern China, in December 2021. Our study emphasizes the urgent need to establish comprehensive and nationwide surveillance of DPV among poultry.

**Abstract:**

Duck plague (DP) is a highly contagious viral disease in ducks caused by the duck plague virus (DPV). The DPV, a member of *Herpesviridae*, poses a severe threat to the waterfowl farming industry worldwide. In this study, we reported a recent outbreak of DPV in domestic laying ducks at 310 days of age from southern China in December 2021. The gross lesion, histopathologic examination, molecular detection, and genetic characterization studies of DPV are described here. As a result, gross lesions such as an enlarged congestive spleen and liver were observed. Liver with vacuolar degeneration and small vacuoles and spleen with hemosiderosis were remarkable microscopic findings. Our results suggested that the liver had the highest viral load, followed by the trachea, pancreas, kidney, brain, spleen, and heart. In addition, DPV was successfully isolated in chicken embryo fibroblast cell culture and designated as DP-GD-305-21. The *UL2*, *UL12*, *UL41*, *UL47*, and *LORF11* genes of DP-GD-305-21 shared a high nucleotide homology with the Chinese virulent (CHv) strain and the Chinese variant (CV) strain. In conclusion, this study reports the isolation and molecular characterization of DPV from a recent outbreak in southern China. Our results contributed to the understanding of the pathological and molecular characterization of currently circulating DPV in China.

## 1. Introduction

Duck plague (DP), also known as duck virus enteritis (DVE), is one of the major highly contagious and lethal diseases in domestic and wild ducks, swans, geese, and other Anseriformes [1,2,3]. DP is caused by the duck plague virus (DPV), which belongs to the genus *Mardivirus*, subfamily *Alphaherpesvirinae*, family *Herpesviridae*, order *Herpesvirales*. DP causes serious digestive mucosal lesions in the oral cavity, heart, intestinal tract, spleen, esophagus, thymus, and bursa of Fabricius [4,5,6]. DP was first reported in the Netherlands in 1923 [7]. Since then, DP has been reported globally, including in China, Poland, Bangladesh, and India, which has severely harmed the global duck breeding industry [6,8,9,10,11]. In China, DP was first reported by Huang et al. in 1957 [12], after which it quickly spread throughout this Asian country. Several cases of DPV were reported in the Shandong province of China in 2012 [6]. The prevalence of DPV in the waterfowl population in southern China was reported to be approximately 3.8% [13].

The mature DPV virions were round, with a diameter ranging from 150 to 300 nm. The genome of DPV consists of a linear double-stranded DNA of approximately 160 kb that contains 78 open reading frames (ORFs) [11]. The highly conserved DPV genome is not affected by the origin or virulence of the strain [14]. The genomes of virulent strains and attenuated strains are distinguished by the unique long (UL) segment with insertions, which are located mainly in the *UL2*, *UL12*, *UL41*, *UL47*, and *LORF11* (left open reading frame 11) genes [11,14,15,16,17]. Currently, the chicken embryo-adapted live DP vaccine is poorly immunogenic and provides only partial protection against DPV [18,19,20]. Several DNA vaccines were developed against DP. DNA vaccines that encode DPV glycoproteins D and B induced a strong response to DPV [21]. Using CRISPR/Cas9, a recombinant C-KCE-HA/PrM-E vaccine was constructed and it can provide protection against DPV, duck Tembusu virus (flavivirus), and highly pathogenic avian influenza virus [22]. Although great effort has been made to develop novel vaccines against DPV, current vaccines for DP remain suboptimal and sporadic outbreaks of DPV in ducks are not uncommon [11].

In this study, we report the pathological and molecular characterization of a DPV outbreak in domestic laying ducks from southern China in December 2021. DPV was isolated and genetic characterization of virulence genes including *LORF11*, *UL2*, *UL12*, *UL41*, and *UL47* was carried out.

## 2. Materials and Methods

### 2.1. Ethics Statement

This study was approved by the Research Ethics Committee of the College of Life Science and Engineering, Foshan University (protocol code 20211818). All ducks were humanely handled in accordance with the protocols and principles of animal ethics. The veterinarians obtained written consent from the owners to collect samples.

### 2.2. Case Report and Sample Collection

In December 2021, a suspected outbreak of DPV occurred on a farm with 5000 hemp ducks at approximately 310 days of age in Qingyuan city, Guangdong, China. Clinical signs of depression, inability to stand, inappetence, white-green diarrhea, watery dejecta, corneal opacity, and sudden death were observed. The number of sick ducks increased daily by 15–30, with a mortality rate of about 30%. There was no obvious effect after treatment with antibiotic drugs. Ducks suspected of having died from DPV infection and those showing typical clinical signs of DP were collected. Tissues from the heart, liver, spleen, kidney, and pancreas (0.5 g) were collected in 1 mL of phosphate-buffered saline (PBS) (pH = 7.4). The tissues were then homogenized with glass beads for 2 cycles at 6000 rpm for 10 s using a Precellys Evolution Super Homogenizer (Bertin Technologies, France). The homogenates were centrifuged for 5 min at 12,000 rpm and the supernatant was collected and stored at −80 °C.

### 2.3. Histopathologic Examination

For histopathological examination, the heart, liver, spleen, kidney, and pancreas tissues were fixed with 10% buffered formalin phosphate (Biosharp, Hefei, China) for 72 h. Fixed tissues were embedded in paraffin and hematoxylin–eosin staining was performed. Each tissue was trimmed to a size of 1.5 × 1.5 × 0.4 cm and kept in the dehydrator (DIAPATH, Bergamo, Italy) to dehydrate with a gradient of alcohol (Sinopharm Group Chemical Reagent Co., Ltd., Beijing, China). The tissues were then soaked in melting paraffin at 65 °C and embedded in the embedding machine (Wuhan Junjie Electronics Co., Ltd., Wuhan, China). After cooling on a −20 °C freezing table, the wax blocks were removed from the embedded frame and trimmed. Trimmed wax blocks were placed on a freezing stage at −20 °C, and cooled tissue section wax blocks were sectioned at 60 °C.

After water drying and melting, wax sections were placed in Xylene I, Xylene II, 100% ethanol I, 100% ethanol II, and 75% ethanol for 5 min. Sections were rinsed with tap water and stained with hematoxylin solution (Servicebio, Wuhan, China) for 3–5 min. The tissue sections were then treated with Hematoxylin Scott Tap Bluing and rinsed with tap water. After being watered, sections were placed in 85% ethanol for 5 min; 95% ethanol for 5 min; eosin dye for 5 min; 100% ethanol I for 5 min; 100% ethanol II for 5 min; 100% ethanol III for 5 min; Xylene I for 5 min; and Xylene II for 5 min. Finally, sections were sealed with neutral gum (Shanghai Clinical Research Center (SCRC), Shanghai, China). The sections were observed and photographed with an upright optical microscope (Nikon, Tokyo, Japan).

### 2.4. DNA Extraction and Virus Detection

The AxyPrep Body Fluid Viral DNA/RNA Miniprep Kit (Axygen, Hangzhou, China) was used to extract the viral DNA from the supernatant of the tissue homogenate following the manufacturer’s instructions. For clinical diagnosis, the partial *UL6* gene was amplified in a 25 μL reaction mixture: 12.5 μL of 2 Taq Plus Master Mix II (Vazyme, Nanjing, China), 1 μL of each primer (DVEV-F: GAGCGTATTTAGTAGAAACTGC; DVEV-R: TGAATGTTGTGATTGTTC): (10 µM) [23], 9 μL of nuclease-free water, and 2 μL of template DNA. The target *UL6* gene was amplified under the following conditions: initial denaturation at 95 °C for 5 min; 35 cycles of denaturation at 95 °C for 30 s; annealing at 53 °C for 30 s; and extension at 72 °C for 30 s. The final extension was performed for 10 min at 72 °C. The PCR products were analyzed on a 2% agarose gel at 120 V for 30 min. Specific fragment bands were extracted using the FastPure Gel DNA Extraction Mini Kit (Vazyme, Nanjing, China). The PCR products were subcloned into the pMD 18-T vector (Takara, Dalian, China) and sequenced using Sanger sequencing (Sangon Biotech, Guangzhou, China).

### 2.5. Virus Titration

A total of 0.35 g of each tissue, including the heart, liver, spleen, kidney, and pancreas, were collected and subjected to viral DNA extraction using the AxyPrep Viral DNA/RNA Miniprep Kit (Axygen). Quantitative real-time PCR was used to determine viral load in each tissue with the following conditions: 5 μL PowerUpTM SYBR^TM^ Green Master Mix (Thermo Fisher Scientific, Waltham, MA, USA), 0.4 μL of each primer (10 μM) [24], 3.2 μL of nuclease-free water, and 1 μL of template DNA. The PCR parameters were set as follows: UDG activation at 50 °C for 2 min; initial denaturation at 95 °C for 2 min; 40 cycles of denaturation at 95 °C for 15 s; annealing at 53 °C for 15 s; and extension at 72 °C for 1 min.

### 2.6. DPV Isolation

Chicken embryo fibroblasts (CEF) tissue culture was prepared from 9-day-old embryos and propagated in DMEM (Thermo Fisher Scientific) with 10% fetal bovine serum (FBS) (Gibco, NY, USA). CEF was grown to form monolayer cells and then infected with 100 µL of diluted tissue homogenate. Cells were freeze-thawed three times when cytopathic changes reached 50% and then centrifuged at 1000 rpm for 5 min. The supernatant was collected, and the viral titer was determined by quantitative real-time PCR.

### 2.7. Amplification of Partial Virulence Sequences

A set of primers composed of *UL2*-F: 5-ATGACAGAACCTGCCACGGAAAC-3 and *UL2*-R: 5-TTATACTGTTCCACAAGGAAGTTGC-3 was designed to amplify the complete *UL2* gene (1002 bp) according to the *UL2* gene sequences of DPV strains deposited in GenBank. Furthermore, the *UL12*, *UL41*, *UL47*, and *LORF11* genes with an expected size of 1689 bp, 1494 bp, 2364 bp, and 4341 bp, respectively, were amplified with the primers described by Wang et al. [25]. These genes were amplified in a 25 μL reaction: 12.5 μL 2 × Taq Plus Master Mix II (Vazyme, Nanjing, China), 1 μL of each primer (10 µM), 9 μL of nuclease-free water (Servicebio, Wuhan, China), and 2 μL template DNA. The PCR parameters were as follows: initial denaturation at 95 °C for 5 min; 35 cycles of denaturation at 95 °C for 30 s; annealing at 52 °C for 30 s; and extension at 72 °C (*LORF11* for 3 min 40 s, *UL12* for 2 min, *UL41* for 1 min 30 s, and *UL47* for 2 min 30 s). The final extension was kept at 72 °C for 10 min. The PCR products were purified using the FastPure Gel DNA Extraction Mini Kit (Vazyme) according to the manufacturer’s instructions. The purified PCR products were subcloned into the pMD 18-T vector (Takara) and then sequenced by Sanger sequencing.

### 2.8. Phylogenetic Analysis

The sequences of *UL2*, *UL12*, *UL41*, *UL47*, and *LORF11* genes from representative DPV strains were retrieved from GenBank database. Pairwise alignment was carried out using the built-in Clustal V program of MEGA 5.1 software. The phylogenetic trees were built using the maximum likelihood (ML) method with 1000 bootstrap replicates.

### 2.9. Bioinformatics Analysis

Secondary structures of proteins such as LORF11, UL2, UL12, UL41, and UL47 were predicted using the Chou–Fasman, Garnier–Robson tools of DNAstar 7.1 software (version 7.1.0) (DNASTAR Inc., Madison, WI, USA). The flexibility regions of the proteins were predicted using the Karplus-Schultz tool; the hydrophilicity of the proteins was predicted using the Kyte–Doolittle tool; the antigenic index of the proteins was predicted using the Jameson–Wolf tool; and the surface probability of the proteins was predicted using the Emini tool. The hydrophobicity of the protein was analyzed by Prot Scale software (https://web.expasy.org/protscale/, accessed on 1 August 2022).

### 2.10. Statistical Analysis

Statistical analyses were performed with the GraphPad Prism 8 software (Version 8.0.2) (GraphPad Software Inc., San Diego, CA, USA). The two-way ANOVA method was used to calculate the *p* values. Data were shown as the mean ± standard deviation. The level of statistical significance was established at *p* < 0.05.

## 3. Results

### 3.1. Gross Lesions

**Figure 1:**
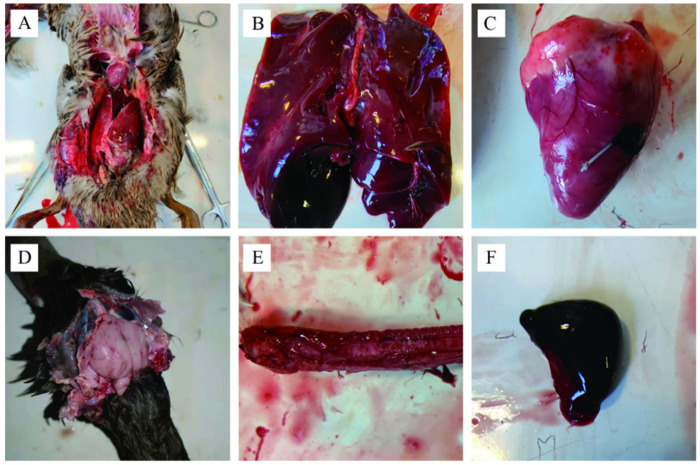
Gross lesions of ducks infected with DPV at autopsy. (**A**): Presence of bold clots related to the pericardial sac and hepatic capsule; (**B**): Enlarged and congested liver; (**C**): Spotted hemorrhage in the heart; (**D**): Enlarged and congested brain; (**E**): Spotted hyperemia of the trachea; (**F**): Enlarged and severely congested spleen.

Grossly, lesions such as congested and enlarged liver (Figure 1A,B), congested and epicardial spotted hemorrhage in the heart (Figure 1C), light edema of the meninges and mildly congested meningeal vessels in the brain (Figure 1D), diffuse hemorrhagic foci in the tracheal mucosa (Figure 1E), and severely enlarged and congested spleen were observed (Figure 1F).

### 3.2. Virus Detection

Amplificaiton of the target *UL6* gene fragment with a size of 416 bp was observed in 2% agarose gel electrophoresis (Supplementary Figure S1). The result of sequence alignment showed that the partial *UL6* gene shared a homology of 98.2% with the *UL6* gene of CV (JQ673560.1), CHv (JQ647509.1), and C-KCE (KF263690.1) strains (Supplementary Figures S2 and S3).

### 3.3. Histopathological Analysis

**Figure 2:**
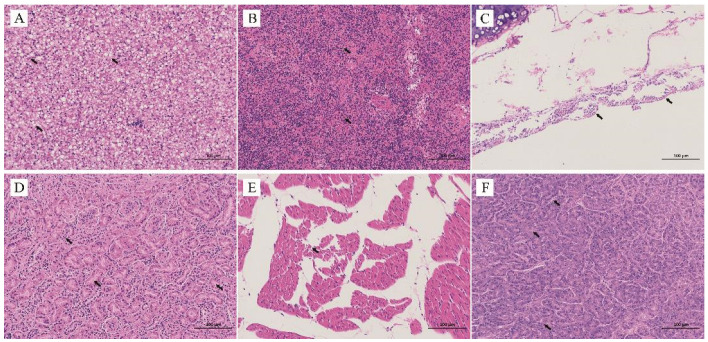
Histopathological images of duck tissue sections stained with hematoxylin and eosin (H&E) (magnification, ×200). The scale bar included in each image presents a length of 100 μm. (**A**): Extensive vacuolar degeneration and small vacuoles were detected in hepatocytes; (**B**): Lymphoid depletion, focal necrosis, and multifocal fibrinoid necrotic foci were observed in spleen; (**C**): Detached epithelial cells and damaged bronchial wall were observed in the tracheal mucosa; (**D**): Generalized tubular atrophy and inflammatory cell infiltration in the interstitium were observed in kidney; (**E**): Cardiomyocytes were tightly packed and surrounded by inflammatory cell infiltration in heart; (**F**): Necrosis of acinar and islet structures were detected in pancreatic tissue.

Extensive vacuolar degeneration of hepatocytes narrowing neighbor sinusoids was detected in hepatocytes (Figure 2A). Lymphoid depletion, focal necrosis, and multifocal fibrinoid necrotic foci were observed in the spleen tissue specimen (Figure 2B). The organizational structure of the bronchial wall of the tracheal mucosa was damaged and the mucosal epithelial cells were necrotic, disintegrated and detached (Figure 2C). Generalized tubular atrophy and inflammatory cell infiltration in the interstitium were observed in kidney tissue (Figure 2D). The cardiomyocytes were tightly packed and surrounded by inflammatory cell infiltration in the heart tissue (Figure 2E). Necrosis of the acinar and islet structures was observed in the pancreatic tissue (Figure 2F).

### 3.4. DPV Isolation

**Figure 3:**
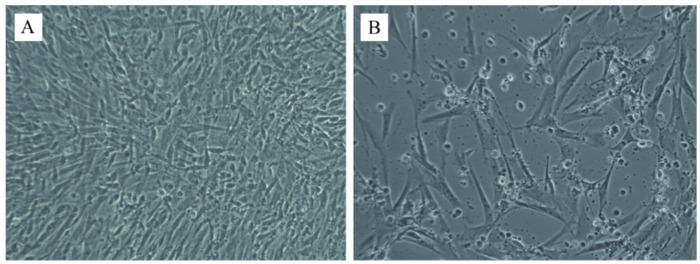
Cytopathic effects on chicken embryo fibroblasts from homogenate duck-infected DPV tissue samples. (**A**): Negative control (100×); (**B**): Infected CEF cells showing vacuolation and syncytia (400×).

A strain of DPV (DP-GD-305-21) was successfully isolated from liver tissues in CEF. After 48 h of infection, the cytopathic effect (CPE) such as pyknotic rounding was found in the CEF at 48 h post-infection. Small grape-like clusters were observed in DPV-infected CEF cells after 48–72 h of infection (Figure 3B). The presence of DPV in CEF was further determined by quantitative real-time PCR.

### 3.5. Viral Load in Tissues

**Figure 4:**
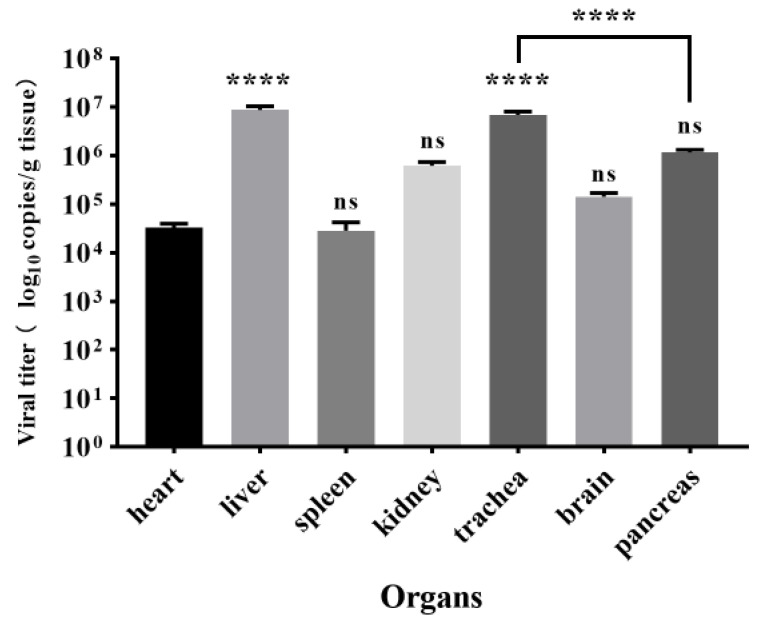
Levels of DPV viral load at the time of initial sampling. The collected tissues are shown at the bottom. Viral loads were determined by quantitative Real-time PCR. Viral loads are expressed as mean ± SD. Differences between groups were determined using two-way ANOVA. ****, *p* < 0.0001; ns, *p* > 0.05.

The viral load in the tissues of the heart, liver, spleen, kidney, trachea, brain, and pancreas was determined by quantitative real-time PCR (Figure 4). Liver tissue reached the highest viral titers of 8.7 × 10^6^ copies/g, which was significantly higher (*p* < 0.0001) than the heart, spleen, kidney, and brain tissues. The heart had the lowest viral load, 3.2 × 10^5^ copies/g, which was not significantly different from the spleen, kidney, and brain (*p* > 0.05).

### 3.6. Sequence Analysis of Virulence Genes

The sequencing results suggested that virulence genes including *UL2*, *UL12*, *UL41*, *UL47*, and *LORF11* were all successfully amplified. The mutations in *UL12*, *UL41*, *UL47*, and *LORF11* of DP-GD-305-21 compared with other representative strains are summarized in Table 1. Results of the phylogenetic analysis suggested that the DP-GD-305-21 had a strong association with the Chinese virulent (CHv) strain and the Chinese variant (CV) strain (Supplementary Figure S4). Detailed analysis of each gene is shown as follows:

#### 3.6.1. Analysis of the UL2 Gene

**Figure 5:**
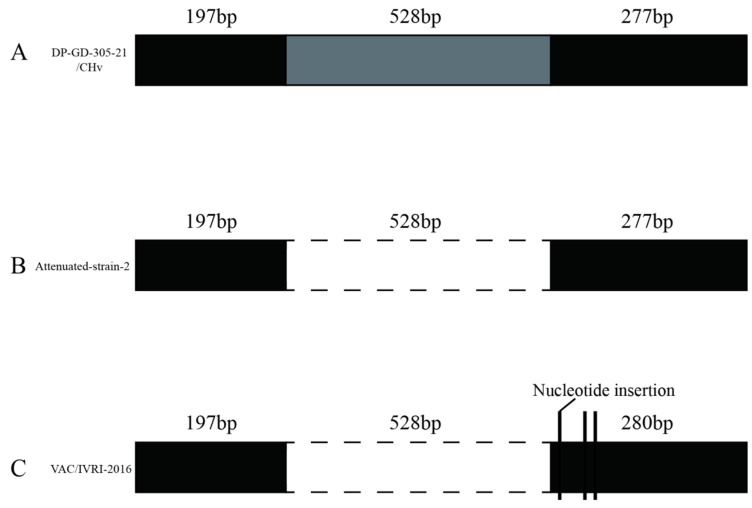
Gene structure types of UL2. (**A**): Strains with a 1002 bp coding region, such as the DP-GD-305-21 and CHv strain; (**B**): Attenuated strain with 528 bp deletion 2; (**C**): Strains with a 528 bp deletion and three 1 bp insertions, including VAC and IVRI-2016.

The length of the *UL2* coding region was 1002 bp. Our results showed that DP-GD-305-21 has a high similarity with strains CHv (JQ647509.1), 2085 (JF999965.1), and CV (JQ673560.1). The homology of DP-GD-305-21 *UL2* with the CHv, CV, LH2011 (KC480262.1), and 2085 strains was 100%. However, it showed a significant difference with the VAC (EU082088.2) and IVRI-2016 (MZ824102.1) strains. Compared to attenuated strain 2, the *UL2* gene of DP-GD-305-21 had a large nucleoside insert of 528 bp (Figure 5). The VAC and IVRI-2016 strains had three base insertions after the 528-base deletion, resulting in 18 consecutive amino acid changes. This deletion did not occur in the attenuated strains.

#### 3.6.2. Analysis of the UL12 Gene

The *UL12* gene of DP-GD-305-21 is 1689 bp long, coding for 562 aa. Compared with other strains in the NBCI GenBank, the *UL12* gene of DP-GD-305-21 had 99.9% aa identity with the strains CHv (JQ647509.1), CV (JQ673560.1), DP-AS-Km-19 (MZ574076.1), C-KCE (KF263690.1), and 2085 (JF999965.1), with a change in aa at position 368. As a result, the 368th amino acid in the *UL2* of DP-GD-305-21 is S instead of A. The *UL12* gene of VAC (EU082088.2) and IVRI-2016 (MZ824102.1) strains codes for 483 aa. The number of clone-03 coding amino acids is 446. This is due to a 236 bp nucleotide deletion at the 5′ end in the *UL12* of VAC and IVRI-2016 strains, and a 349 bp deletion at the 5′ end of the clone-03 strain.

#### 3.6.3. Analysis of the UL41 Gene

The DPV DP-GD-305-21 *UL41* is 1494 bp long, coding for 497 amino acids. It shares 99.6% similarity with strains 2085 (JF999965.1), DP-AS-Km-19 (MZ574076.1), and IVRI-2016 (MZ824102.1), and 99.4% similarity with strains CHv (JQ647509.1), C-KCE (KF263690.1), and VAC (EU082088.2). In the *UL41* gene of DP-GD-305-21, the deletion of the amino acids was located at position 125, which is the same as the SD (MN518864.1), LH2011 (KC480260.1), and 2085 strains.

#### 3.6.4. Analysis of the UL47 Gene

The sequencing results showed that the length of the DP-GD-305-21 *UL47* fragment is 2364 bp. The sequence of strain DP-GD-305-21 *UL47* shares the highest homology (99.9%) with that of strains 2085 (JF999965.1) and DP-AS-Km-19 (MZ574076.1), the difference being the three nucleotide variants in DP-GD-305-21. Compared with the Cv (JQ673560.1), CHv (JQ647509.1), VAC (EU082088.2), and clone-03 (EF524094.1) strains, a three-base deletion was found at position 445 in DP-GD-305-21, which is the same as strains 2085, DP-AS-Km-19 and IVRI-2016 (MZ824102.1), resulting in DP-GD-305-21 having one amino acid less than these strains.

#### 3.6.5. Analysis of the LORF11 Gene

**Figure 6:**
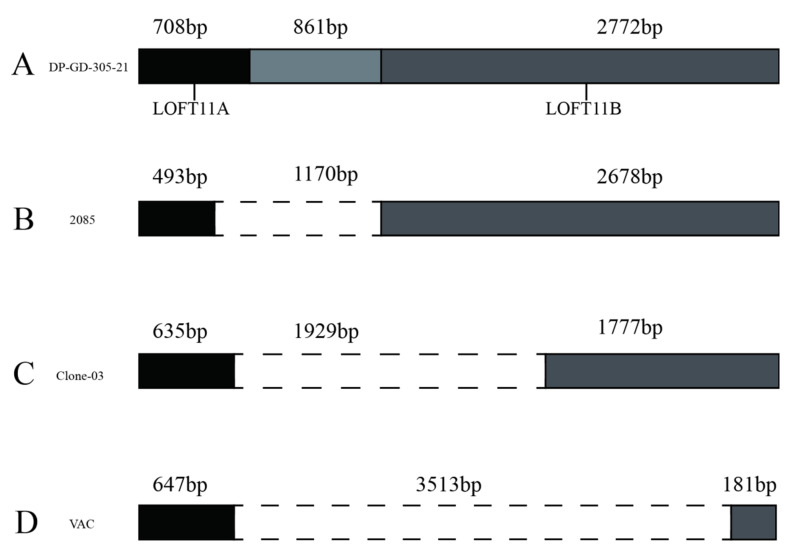
Gene structure types of LOFT11. (**A**): A coding region of 4341 bp for the LOFT11 gene was observed in strains such as DP-GD-305-21; (**B**): Strains such as the 21 have a 1170 bp deletion in the coding region of LORF11; (**C**): A 1929 bp deletion was found in the LOFT11 gene of strains such as the ‘Clone-03’; (**D**): A 3513 bp deletion was found in the LOFT11 gene of strains such as the VAC strain.

The DP-GD-305-21 *LORF11* coding region is 4341 bp long, coding for 1445 aa. Compared with other strains, the *LORF11* gene can be divided into four types (Figure 6). In contrast, the insertion sequence in the middle of the 2085 (JF999965.1), DP-AS-Km-19 (MZ574076.1), D11-JW-016 (JQ430739.1), Holland (JQ595417.1), DP-GD-305-21, Cv (JQ673560.1), and CHv (JQ647509.1) strains is 1170 bp. Their *LORF11* is divided by this insertion into two ORFs, *LORF11A* and *LORF11B*. A 493 bp sequence in the 5′ *LORF11* of the 2085, DP-AS-Km-19, D11-JW-016, and Holland strains showed high homology with *LORF11A* of DP-GD-305-21. The clone-03 (EU294364.1) strain *LORF11* sequence had a 1929 bp deletion in the middle, and 635 bp and 1777 bp sequences at both ends showed high homology with the *LORF11A* and *LORF11B* of the DP-GD-305-21, respectively. The IVRI-2016 (MZ824102.1) and VAC (EU082088.2) strains have a deletion of 3513 bp in the middle of the *LORF11* gene. A 142 bp fragment was found in Chinese strains, including virulent strains and attenuated strains, located at the 494th position of the *LORF11* gene.

### 3.7. Prediction of the Protein Structure

**Figure 7:**
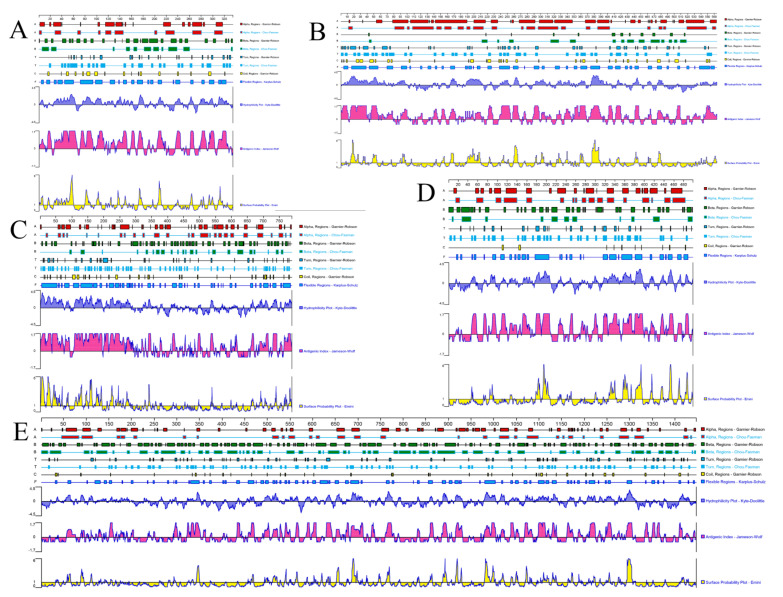
The secondary structure, hydrophilicity, flexible regions, antigenic index, and surface probability of the DP-GD-305-21 proteins. (**A**): UL2 protein; (**B**): UL12 protein; (**C**): UL47 protein; (**D**): UL41 protein; (**E**): LORF11 protein.

The secondary structures of the *UL2*, *UL12*, *UL41*, *UL47*, and *LORF11* proteins were predicted by the Garnier–Robson and Chou–Fasman methods of DNAstar software. The number of alpha, helix, and beta-collapse regions in these proteins was calculated (Supplementary Table S1 Corner of the region, Random-coil region, Flexible region). The hydrophilicity, flexible regions, antigenic index, surface probability, and Hydrophobic prediction of *UL2*, *UL12*, *UL41*, *UL47*, and *LORF11* proteins are shown in Figure 7 and Supplementary Figure S5.

## 4. Discussion

In this study, the suspected DPV infection of ducks on a farm of hemp ducks in Guangdong, China, in December 2021 was confirmed by clinical signs, autopsy characteristics, histopathological analysis, virus isolation, and virus sequencing. Previously published studies described ducks infected with DPV that showed depression, lethargy, dehydration, watery dejecta, and nasal discharge [26], which is consistent with the results of this study. Additionally, clinical signs such as lacrimation were observed in the sick ducks in this study. The mortality rate (30%) in the present case was similar to an outbreak among Australian black swans that happened in 2018 [9], while Pazhanivel et al. recorded a high mortality of 44.4% in ducks [4]. Mallard ducks were suggested to be less susceptible to DPV and bird mortality varies between duck species [11].

Gross lesions such as the presence of bold clots related to the pericardial sac and hepatic capsule, congested liver, and congestion of meningeal blood vessels in the brain were observed in DPV-infected ducks, which is consistent with the results of earlier studies [27,28,29]. Our results suggested that the liver and trachea had the highest viral load, while the heart had the lowest viral load. On the contrary, a previous study by Kumar et al. showed that the spleen and liver had the highest viral loads [30]. This variation may be caused by the fact that they did not check the viral load in the trachea. DPV was suggested to be adapted to CEF and duck embryo fibroblasts (DEF) [11]. Similar to previous studies [31], DPV was isolated by propagating in CEF, and CPE was observed after 72 h of infection.

DPV genomes from different countries were suggested to share a high homology [32]. However, DPV can be classified into virulent strains and attenuated strains based on the length of some virulent-associated genes. For example, *UL2*, which is predicted to encode a uracil DNA glycosylase and play an important role in viral replication, showed a significant difference between virulent strains and attenuated strains [15,33]. Nevertheless, a recent report suggested that the deletion in UL2 was not related to the attenuated virulence of the DPV vaccine strain [34]. In this study, the analysis of the *UL2* gene of the strain DP-GD-305-21 showed high homology with virulent strains and a significant difference with attenuated strains. In contrast to the attenuated strain, there was a 528 bp insertion in the middle of the *UL2* gene of the DP-GD-305-21. The *UL12* gene of DP-GD-305-21, coding for 562 aa, is the same as that of the virulent strains, such as CHv, LH2011, ZJ2016, and 2085. However, the *UL12* gene of the clone-03 strain and the VAC strain code for 483 aa and 446 aa, respectively.

The structure of the *LORF11* gene in the DP-GD-305-21 strain was similar to that of the CHv, SD, and CV strains. The *LORT11* gene of the DP-GD-305-21 strain has a 142 bp sequence after LORF11A, which is similar to Chinese traditional strains. Interestingly, this 142 bp fragment was not found in the *LORT11* gene of foreign strains. The results suggested that this 142 bp fragment may be related to the region of the virus and is specific to the Chinese strain. Taken together, the *UL2*, *UL12*, *UL41*, *UL47*, and *LORF11* genes of DP-GD-305-21 share the same characteristics as the virulent strain. The results of the sequence analysis of the *LORF11* gene of DP-GD-305-21 indicated that DP-GD-305-21 is a domestic virulent strain.

## 5. Conclusions

In summary, this study reported a recent outbreak of DPV on a farm in Guangdong, China. The DPV was isolated and the novel genetic characterization of the *LORF11*, *UL2*, *UL12*, *UL41*, and *UL47* virulence genes was determined. Our study would help improve the understanding of the pathogenesis of DPV circulating in southern China and highlights the urgent need for enhanced DPV surveillance among poultry to avoid devastating economic losses and improve prevention and control measures.

## Figures and Tables

**Table 1:** Point mutation in *UL12*, *UL41*, *UL47*, and *LORF11* of DP-GD-305-21, compared with other representative strains. * Represents deletion.

ORF	Amino Acid Position(s)	Amino Acid						
		DP-GD-305-21	CHv (JQ647509.1)	CV (JQ673560.1)	SD (JQ673560.1)	LH2011 (MZ574076.1)	2085 (JF999965.1)	DP-AS-Km-19 (MZ574076.1)
*UL12*	368	S	A	A	A	A	A	A
*UL41*	125	- *	D	D	-	-	-	D
	303	Y	Y	Y	Y	C	C	C
*UL47*	113	H	R	R	H	H	H	H
	149	-	D	D	-	-	-	-
	404	A	V	V	V	V	V	V
	599	A	T	T	A	A	A	A
	727	F	L	L	L	L	L	L
*LORF11*	2	-	E	E	E	A	E	E
	435	S	P	P	P	P	-	-
	486	K	N	N	K	N	-	-
	713	I	V	V	I	V	I	I
	745	R	H	H	R	H	R	R
	789	Y	-	-	Y	-	Y	Y
	869	L	R	L	L	L	L	L
	894	R	H	H	R	H	R	R

## Data Availability

The data generated in this study are available from the corresponding author upon reasonable request.

## Supplementary Materials

The following supporting information can be downloaded at: https://www.mdpi.com/article/10.3390/ani12243523/s1. Figure S1. PCR product of the fragment of the DPV partial UL6 gene detected by 2% agarose gel electrophoresis. Figure S2. Alignment of the DPV partial UL6 gene with other strains. Figure S3. Homology analysis of the DPV partial UL6 gene with other strains. Figure S4. Phylogenetic analysis of *UL2*, *UL12*, *UL41*, *UL47*, and *LORF11* gene of DEV strain (DP-GD-305-21) (marked with a triangle) collected in this study. Figure S5. Hydrophobic prediction of the DP-GD-305-21 proteins. A: UL2 protein; B: UL12 protein; C: UL41 protein; D: UL47 protein; E: LORF11 protein. Table S1, Alpha helix and beta-collapse region in *UL2*, *UL12*, *UL41*, *UL47*, and *LORF11* proteins of DP-GD-305-21.
