# Supplementary material for: Pathological and Molecular Characterization of a Duck Plague Outbreak in Southern China in 2021
Source: Animals (Basel). 2022 Dec 13;12(24):3523. doi: 10.3390/ani12243523 (PMC9774102; doi:10.3390/ani12243523)
Supplement: Supplementary file 1 [file animals-12-03523-s001.zip › animals-1999919-supplementary (1).pdf]

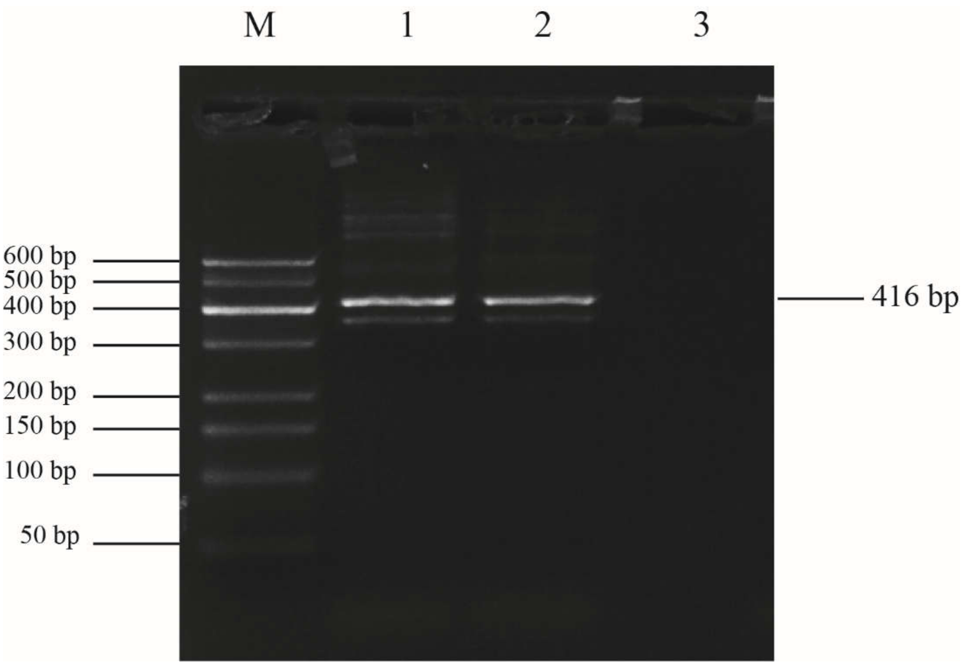

Supplementary Figure S1. PCR product of the fragment of the DPV partial UL6 gene detected by 2% agarose gel electrophoresis. M: DNA marker; **1**: PCR product of the DPV partial UL6; **2**: PCR product of the DPV partial UL6; **3**: Negative control;

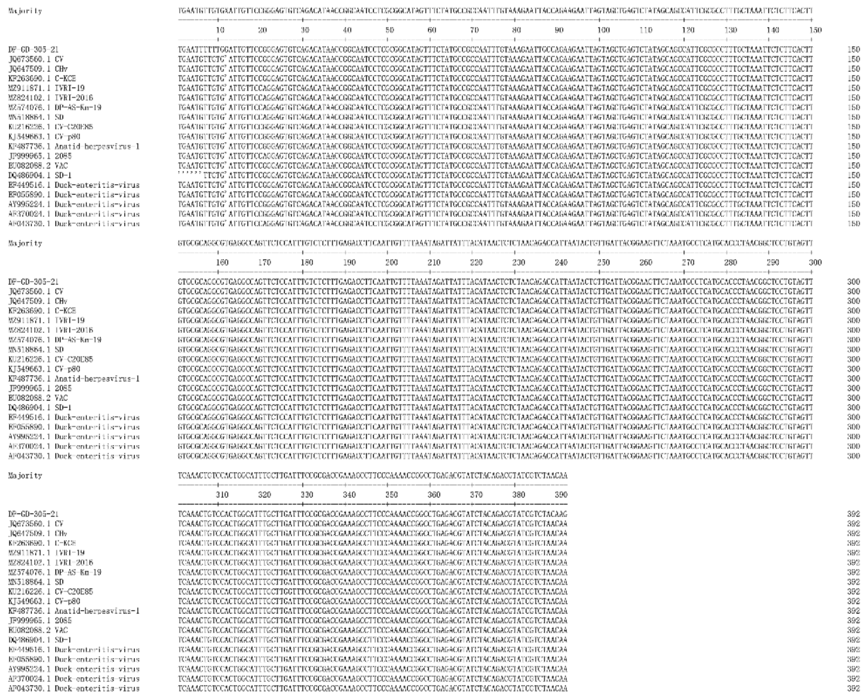

Supplementary Figure S2. Alignment of the DPV partial UL6 gene with other strains.

Percent Identity

Divergence

	1	2	3	4	5	6	7	8	9	10	11	12	13	14	15	16	17	18	19		
1		98.2	98.2	98.2	98	98	98	98	98	98.2	98.2	98	98.2	96.9	98.2	98.2	98.2	98.2	98	1	DP-GD-305-21
2	1.6		100	100	99.7	99.7	99.7	99.7	99.7	100	100	99.7	100	98.5	100	100	100	100	99.7	2	JQ673560.1 CV
3	1.6	0		100	99.7	99.7	99.7	99.7	99.7	100	100	99.7	100	98.5	100	100	100	100	99.7	3	JQ647509.1 CHv
4	1.6	0	0		99.7	99.7	99.7	99.7	99.7	100	100	99.7	100	98.5	100	100	100	100	99.7	4	KF263690.1 C-KCE
5	1.8	0.3	0.3	0.3		100	100	100	99.5	99.7	99.7	100	99.7	98.2	99.7	99.7	99.7	99.7	100	5	MZ911871.1 IVRI-19
6	1.8	0.3	0.3	0.3	0		100	100	99.5	99.7	99.7	100	99.7	98.2	99.7	99.7	99.7	99.7	100	6	MZ824102.1 IVRI-2016
7	1.8	0.3	0.3	0.3	0	0		100	99.5	99.7	99.7	100	99.7	98.2	99.7	99.7	99.7	99.7	100	7	MZ574076.1 DP-AS-Km-19
8	1.8	0.3	0.3	0.3	0	0	0		99.5	99.7	99.7	100	99.7	98.2	99.7	99.7	99.7	99.7	100	8	MN518864.1 SD
9	1.8	0.3	0.3	0.3	0.5	0.5	0.5	0.5		99.7	99.7	99.5	99.7	98.2	99.7	99.7	99.7	99.7	99.5	9	KU216226.1 CV-C20E85-isolate-passage-85-on-CE
10	1.6	0	0	0	0.3	0.3	0.3	0.3	0.3		100	99.7	100	98.5	100	100	100	100	99.7	10	KJ549663.1 CV-p80
11	1.6	0	0	0	0.3	0.3	0.3	0.3	0.3	0		99.7	100	98.5	100	100	100	100	99.7	11	KF487736.1 Anatid-herpesvirus-1
12	1.8	0.3	0.3	0.3	0	0	0	0	0.5	0.3	0.3		99.7	98.2	99.7	99.7	99.7	99.7	100	12	JF999965.1 2085
13	1.6	0	0	0	0.3	0.3	0.3	0.3	0.3	0	0	0.3		98.5	100	100	100	100	99.7	13	EU082088.2 VAC
14	1.3	0	0	0	0.3	0.3	0.3	0.3	0.3	0	0	0.3	0		98.5	98.5	98.5	98.5	98.2	14	DQ486904.1 SD-1
15	1.6	0	0	0	0.3	0.3	0.3	0.3	0.3	0	0	0.3	0	0		100	100	100	99.7	15	EF449516.1 Duck-enteritis-virus
16	1.6	0	0	0	0.3	0.3	0.3	0.3	0.3	0	0	0.3	0	0	0		100	100	99.7	16	EF055890.1 Duck-enteritis-virus
17	1.6	0	0	0	0.3	0.3	0.3	0.3	0.3	0	0	0.3	0	0	0	0		100	99.7	17	AY995224.1 Duck-enteritis-virus
18	1.6	0	0	0	0.3	0.3	0.3	0.3	0.3	0	0	0.3	0	0	0	0	0		99.7	18	AF370024.1 Duck-enteritis-virus
19	1.8	0.3	0.3	0.3	0	0	0	0	0.5	0.3	0.3	0	0.3	0.3	0.3	0.3	0.3	0.3		19	AF043730.1 Duck-enteritis-virus
	1	2	3	4	5	6	7	8	9	10	11	12	13	14	15	16	17	18	19		

Supplementary Figure S3. Homology analysis of the DPV partial UL6 gene with other strains.

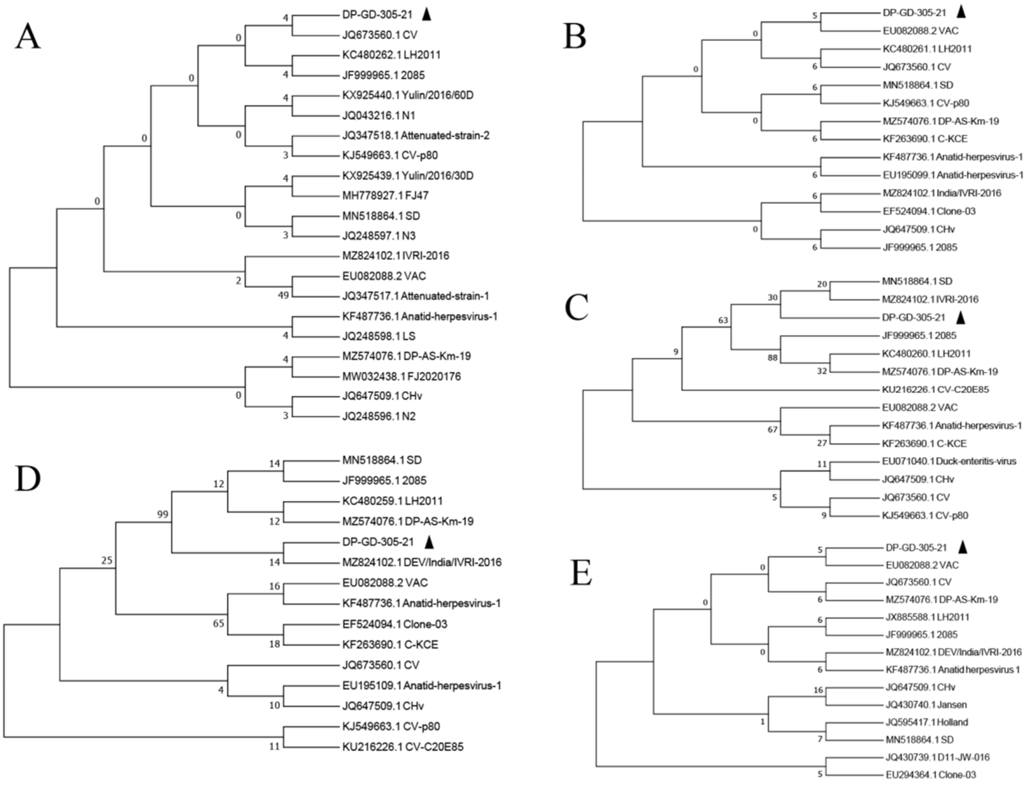

Supplementary Figure S4. Phylogenetic analysis of *UL2, UL12, UL41, UL47,* and *LORF11* gene of DEV strain (DP-GD-305-21) (marked with a triangle) collected in this study. The phylogenetic trees were built using the maximum likelihood (ML) method with 1,000 bootstrap replicates. A: Phylogenetic tree of UL2 gene; B: Phylogenetic tree of UL12 gene; C: Phylogenetic tree of UL41 gene; D: Phylogenetic tree of UL47 gene; F: Phylogenetic tree of LORF11 gene.

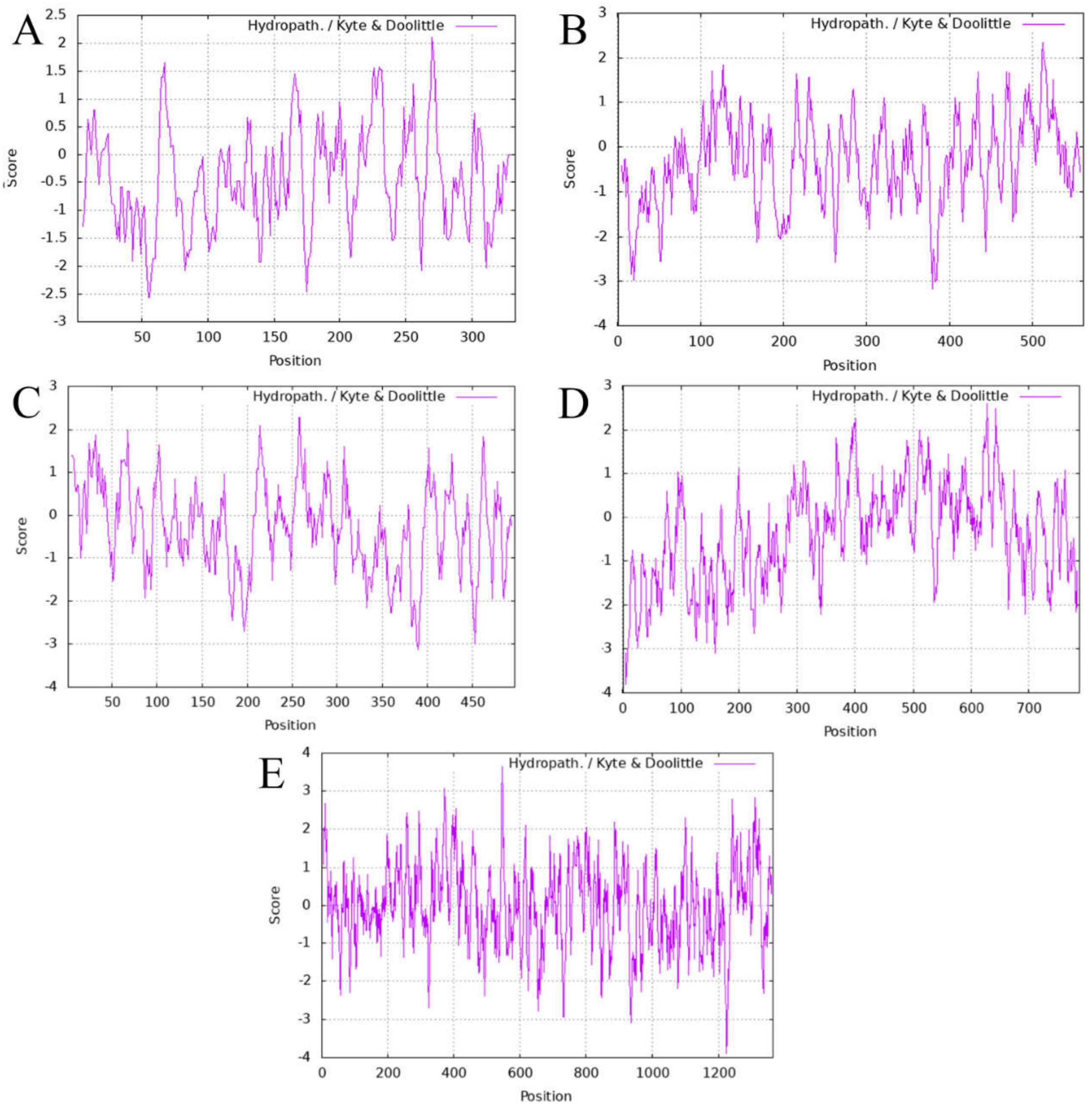

Supplementary Figure S5. Hydrophobic prediction of the DP-GD-305-21 proteins. A: UL2 protein; B: UL12 protein; C: UL41 protein; D: UL47 protein; E: LORF11 protein.

Supplementary Table S1. Alpha helix and beta-collapse region in *UL2, UL12, UL41, UL47*, and *LORF11* proteins of DP-GD-305-21.

**protein**	**method**	
	**Garnier-Robson**	**Chou-Fasman**
UL2	129	91
UL12	33	113
UL41	202	99
UL47	308	179
LORF11	717	692
